# 

**Published:** 1890-01-11

**Authors:** 


SATURDAY, JANUARY 11th, 1890.

**Great and Enduring Deeds** - 223
**Words of Consolation** —Knowing Christ - 224
**Pauperism in London** - 225
**The History and Capabilities of Herbal Simples.**—III.—The Wood-Anemone, Angelica, Aniseed. By W. T. Fernie, M.D. - 226
**The Doctor's Pulpit.**—Nerves and Epidemics - 227
**Everybody's Page** - 228
**The Proposed Registration of Nurses** - 229
**Notes and News** - 230
**Annotations.**—Antiseptics *v.* Plain Water—Missing Contributions to Charities—Directions for Influenza - 232
**A Vade Mecum for New Year's Givers.**—General Hospitals—Fever Hospitals—Hospitals for Children—Miscellaneous - 236
**The Practitioner's Outlook and Retrospect:**
Medicine—Ophthalmia Neonatorum—Tetanus a Communicable Disease—Biliary Calculi and Olive Oil—Treatment of Malarial Fever - 233
Diabetes in Childhood—Antipyrin—Accumulation of Arsenic in Spongy Bone—An Infantile Complication - 234
Surgery—Cancer of the Tongue—Antisepsis—Treatment of Goitre by Iodine - 235
New Drugs, Appliances, and Things Medical—George Mason and Co.'s Concentrated Meat Preparations and O.K. Sauce - 235
**Popular Pastimes as Aids to Hospital Funds.** By A. W. Webster - 237
**Fallow Crops.**—Charity Lotteries - 238
**Scraps and Gleanings** - 238

**EXTRA SUPPLEMENT—THE NURSING MIRROR.**

**En Passant.**—Christmas Cards—Short Items—Nurses at Melbourne—Cottage Nurses—The Strike at Leith—Philadelphia News - lxi
**The Rights and Wrongs of Nursing Uniforms** - lxii
**Keeping Christmas** - lxiii
**Everybody's Opinion.**—Overworked Nurses: A Correction - lxiv
**Presentations—Notes and Queries** - lxiv
**Appointments—Vacancies** - lxiv
**Notice—Off Duty** - lxiv

